# 

**Published:** 1885-01

**Authors:** 


### CONTENTS:

Art. I—A Method of Mounting a Porcelain-Faced Crown. By A. O. Hunt, D. D. S. . 385

Art. II—Proceedings of Odontological Society of Great Britain . . . . . . . . . . . . . . . . 389

Art. III—The Matrix in Amalgam Fillings. By Dr. G. C. Caboll . . . . . . . . . . . . . . . . 393

Art. IV—The Causes and Prevention of Dental Caries. By Henry Sewill, M. R. C. S. . 396

Art. V—Four Cases of Closure of the Jaws, and Treatment. By Christopher Heath, M. R. C. S . . . . . . . . . . . . . . . . 401

Art. VI—The New Local Anæsthetic—Hydrochlorate of Cocaine. By J. Edw. Line, D. D. S . . . . . . . . . . . . . . . . 404

Art. VII—Pulp Capping and Treatment of Pulp Canals. By Dr. J. H. Beebee . . . . . 419

Art. VIII—On Coacine and its Hydrochlorate . . . . . . . . . . . . . . . . 412

Art. IX—Cheoplasty, By Lawrence Vanderpant, L. D. S., etc . . . . . . . . . . . . . . . . 420

Art. X—A New Methoh of Placing the Old-Fashioned Pivot Crown. By Dr. E. C. Redgell . . . . . . . . . . . . . . . . 423

Art. XI—Hand Mortar for Mixing Amalgam. By D. Genese, D. D S . . . . . . . . . . . . . . . . 423

### EDITORIAL, ETC.

The Dental Schools . . . . . . . . . . . . . . . . 424
Dental Education . . . . . . . . . . . . . . . . 425

### BIBLIOGRAPHICAL.

Chapin A. Harris' Principles and Practice of Dentistry. Revised. 11th Edition by Ferdinand J. S. Gorgas, M. D., D. D. S . . . . . . . . . . . . . . . . 426
Transactions of Odontological Society of Pennsylvania . . . . . . . . . . . . . . . . 428

### MONTHLY SUMMARY.

A Novel Filling . . . . . . . . . . . . . . . . 429

Strangled to Death by False Teeth . . . . . . . . . . . . . . . . 430

Muriate of Cocaine . . . . . . . . . . . . . . . . 430

Teeth and Brain . . . . . . . . . . . . . . . . 431

The 17th Annual Meeting of the Southern Dental Association . . . . . . . . . . . . . . . . 433

